# More than semantics: Language, power, and epistemic justice in South African scholarship

**DOI:** 10.4102/jcmsa.v3i1.283

**Published:** 2025-09-29

**Authors:** Clint Hendrikse

**Affiliations:** 1 Department of Family, Community and Emergency Care, Division of Emergency Medicine, Faculty of Health Sciences, University of Cape Town, Cape Town, South Africa

‘Language is never neutral. It carries history, power, and consequence’.

Paulo Freire, Brazilian educator and philosopher

Language does more than describe reality – it shapes it, frames whose knowledge counts, which problems are prioritised, and how solutions are framed. In academic discourse, we often use language without questioning the impact it has on how people or communities are perceived, how knowledge is valued and whose experiences are prioritised. Binaries such as ‘developed versus developing countries’, ‘resource-rich versus resource-limited’, and ‘donors versus beneficiaries’ are frequently used, but are loaded with assumptions and colonial legacies, supporting a worldview in which certain groups are seen as whole and able, while others are seen as lacking or perpetually in need. Even terms like ‘Third World versus First World’, although largely phased out, continue to shape perceptions of global inequality, where only the Global North is seen as knowledgeable, advanced, and innovative.^1^ These labels imply that progress follows a unidirectional path towards Global North ideals. By using language like this, we do not only describe inequality, we help to sustain it.

This pattern of reductive language is particularly evident when our academic discourse reduces Africa to a single homogenous entity, ignoring the vast national, cultural, linguistic and institutional differences of the 54 countries it represents. Phrases like ‘African settings’ and ‘African context’ erase diverse realities, nuances and context, while flattening identities. It often says more about the outsider’s gaze than about the realities on the ground. When we use this terminology uncritically, we diminish local agency and complexity and obliterate local knowledge and innovation.

Linked closely to the homogenisation of African contexts, is the widespread use of the term ‘resource-limited settings’, a label that reduces entire countries or regions to sites of scarcity. This term is applied broadly and often without qualification and reinforces a deficit-based narrative where the focus and emphasis are on what is lacking and not on the existing strengths, local expertise, creativity and institutional knowledge. Van Zyl et al. found this phrase to be vague and inconsistently applied, and more often than not fails to capture the realities and capabilities of each setting and is rarely accompanied by any contextual explanation.^2^ These classifications also do not take into consideration that resource constraints exist globally, including in high-income countries. It perpetuates the narrative that some health systems are inherently dependent on external aid and assistance. Also, what is interpreted as ‘limited’ often reflects benchmarks from high-income settings, where models of best practice are not applicable without accounting for feasibility, or cost-effectiveness, in other contexts.^3^ An abundance mindset, however, challenges us to frame our contexts in terms of what is present and not what is missing, and to consider value, possibility, rather than perpetual need.

The tendency to frame African context in terms of lack is also reflected in how we talk about ‘capacity’. According to Geissler et al., capacity in African health sciences should be understood not as an absence to be filled by external expertise, but as something rooted in history and shaped by local realities.^4^ Terms like ‘capacity building’ can be misleading as they imply deficiency and a one-directional flow of expertise from North to South. It assumes that capacity does not exist locally, thereby overlooking existing skills, infrastructure, and institutional memory. Language like ‘capacity building’ reinforces dependency, while ‘capacity development’ or ‘capacity strengthening’ may suggest more equal partnership.

Central to this discussion is the concept of epistemic justice – the fair recognition of diverse ways of knowing, naming and contributing to knowledge.^5^ Epistemic justice asks us to speak, and to define, from where we stand. This means resisting the pressure to mirror dominant norms and instead defining knowledge on our own terms. Reclaiming voice involves challenging the dominance of certain worldviews and the marginalisation of others.

Reframing academic language is not about censorship, but rather about being intentional. South African scholars should adopt more reflective practices that recognise complexity, respecting human dignity, and question long-standing inherited hierarchies. This involves several key actions:

- Be specific and contextual: Rather than using vague terms like ‘low-resource settings’, describe the actual context – for example, ‘district hospitals in the Western Cape with limited CT-scan capacity’.
- Avoid flattening identities: Instead of generalising with phrases like ‘South African communities’ or ‘African researchers’, refer to specific provinces, communities, linguistic groups, or institutions where relevant.
- Interrogate generalised terms: Avoid reductive binaries such as ‘developed’ and ‘developing’, which obscure structural diversity, reinforce historical hierarchies, and imply a single trajectory of progress. Use more precise alternatives like ‘high-income and low-income settings’, or describe context-specific characteristics instead.

Authors, editors and reviewers all shape the standards of academic writing and play a crucial role in promoting inclusive and context-aware writing. Authors should be encouraged to reflect and use more locally grounded framing, and reviewers and editors can help recognise when authors default to generic, externally driven language. This is not about writing style or stylistic preferences, but rather what is fundamental to justice in knowledge production.

Language reflects values. It shapes what counts as knowledge and who is seen as a knowledge holder. Academic discourse that reflects our histories, values local knowledge, and rejects reductive labels is not simply an act of precision – it is an act of justice. If we are serious about creating an equitable and representative academic landscape, we must begin by being more deliberate with the words we use. A more just academic future begins with the words we choose today.

## References

[1] Khan T, Abimbola S, Kyobutungi C, Pai M. How we classify countries and people – And why it matters. BMJ Glob Health. 2022;7(6):e009704. 10.1136/bmjgh-2022-009704 PMC9185389 35672117

[2] Van Zyl C, Badenhorst M, Hanekom S, Heine M. Unravelling ‘low-resource settings’: A systematic scoping review with qualitative content analysis. BMJ Glob Health. 2021;6(6):e005190. 10.1136/bmjgh-2021-005190 PMC8183220 34083239

[3] Hendrikse C. Adrenaline vs noradrenaline: Equity, context, and the realities of best practice. Afr J Emerg Med. 2025;15(3):100891. 10.1016/j.afjem.2025.100891 40823630 PMC12351318

[4] Geissler PW, Tousignant N. Capacity as history and horizon: Infrastructure, autonomy and future in African health science and care. Can J Afr Stud Rev Can Études Afr. 2016;50(3):349–359. 10.1080/00083968.2016.1267653

[5] Bhakuni H, Abimbola S. Epistemic injustice in academic global health. Lancet Glob Health. 2021;9(10):e1465–e1470. 10.1016/S2214-109X(21)00301-6 34384536

